# Inactivation of Three RG(S/T)GR Pentapeptide-Containing Negative Regulators of HetR Results in Lethal Differentiation of *Anabaena* PCC 7120

**DOI:** 10.3390/life10120326

**Published:** 2020-12-04

**Authors:** Ivan Khudyakov, Grigory Gladkov, Jeff Elhai

**Affiliations:** 1 All-Russia Research Institute for Agricultural Microbiology, 196608 Saint-Petersburg, Russia; ruginodis@gmail.com; 2 Department of Biology, Virginia Commonwealth University, Richmond, VA 23284, USA; elhaij@vcu.edu

**Keywords:** cyanobacteria, heterocyst, regulation of differentiation

## Abstract

The filamentous cyanobacterium *Anabaena* sp. PCC 7120 produces, during the differentiation of heterocysts, a short peptide PatS and a protein HetN, both containing an RGSGR pentapeptide essential for activity. Both act on the master regulator HetR to guide heterocyst pattern formation by controlling the binding of HetR to DNA and its turnover. A third small protein, PatX, with an RG(S/T)GR motif is present in all HetR-containing cyanobacteria. In a nitrogen-depleted medium, inactivation of *patX* does not produce a discernible change in phenotype, but its overexpression blocks heterocyst formation. Mutational analysis revealed that PatX is not required for normal intercellular signaling, but it nonetheless is required when PatS is absent to prevent rapid ectopic differentiation. Deprivation of all three negative regulators—PatS, PatX, and HetN—resulted in synchronous differentiation. However, in a nitrogen-containing medium, such deprivation leads to extensive fragmentation, cell lysis, and aberrant differentiation, while either PatX or PatS as the sole HetR regulator can establish and maintain a semiregular heterocyst pattern. These results suggest that tight control over HetR by PatS and PatX is needed to sustain vegetative growth and regulated development. The mutational analysis has been interpreted in light of the opposing roles of negative regulators of HetR and the positive regulator HetL.

## 1. Introduction

In response to different environmental cues, a subpopulation of vegetative cells from nostocalean cyanobacteria can differentiate into different endpoints: terminally differentiated heterocysts for aerobic nitrogen fixation, suicidal necridia for filament fragmentation to produce motile hormogonia for dissemination, and dormant akinetes for survival in harsh environments [1,2,3,4,5]. The model strain *Anabaena/Nostoc* sp. PCC 7120 (hereafter *Anabaena* PCC 7120) does not produce akinetes and hormogonia in laboratory conditions, although it contains all genes known to be necessary for at least hormogonia production (Figure S5 in [6]). It has been widely used to study different aspects of heterocyst differentiation and nitrogen fixation, including the mechanisms governing what is arguably the simplest and most ancient example of a linear semiregular biological pattern that formed by heterocysts along filaments of vegetative cells. It is evident that despite their apparent simplicity, the regulatory networks underlying heterocyst pattern formation are highly complex and multilayered [3,7].

According to a widely accepted model for the initiation of heterocyst differentiation, the accumulation of 2-oxoglutarate (2-OG) in cyanobacteria under nitrogen deprivation [8] is perceived as a nitrogen starvation signal by the global transcriptional regulator NtcA [9]. Activated by its positive effector 2-OG [10,11,12], NtcA can act as either an activator or repressor of numerous genes [13]. 2-OG-activated NtcA activates the transcription of *nrrA* [14], which, in turn, activates the transcription of *hetR* [15]. HetR is a master regulator of differentiation specific to filamentous cyanobacteria [16]. The expression of multiple heterocyst-specific genes depends on HetR, but only a few have been shown to be regulated directly, and repression of several promoters in vegetative cells has been reported (see [17] for a recent review).

NtcA and HetR constitute a mutually dependent positive regulatory circuit [18]. Transcriptional regulation of *hetR* is complex—it is transcribed from multiple promoters that are regulated both temporally and spatially [19]. There is more than transcriptional regulation, however, as replacement of normal transcriptional control of the chromosomal *hetR* with ectopic expression from a copper-regulated *petE* promoter resulted in a wild-type-like heterocyst patterning, which depended on post-transcriptional regulation of HetR protein levels [20], of which phosphorylation is a major part [21,22].

Proteins from three distinct genes, a small peptide PatS [23], a small protein PatX [16], and HetN protein [24,25], are known to suppress heterocyst differentiation when overexpressed. The only feature they all have in common is an RGSGR motif (a few PatX alleles, including that in *Anabaena* PCC 7120, contain RGTGR instead), which was shown to be essential for suppression of heterocyst formation [23,26]. Both PatS and HetN have been shown to block the positive autoregulation of *hetR* [27,28]. *patS* and *patX* expression is induced early (6–8 h after combined nitrogen deprivation) in regularly spaced cells that will become heterocysts [16,23] from DIF1 motif (TCCGGA)-containing, NtcA- and HetR-dependent promoters [29,30]. While *patS* expression is downregulated in mature heterocysts, expression of *hetN* first localizes to committed heterocysts [27] and continues also after heterocyst maturation [31,32].

Inactivation of *patS* leads initially to a phenotype of multiple contiguous heterocysts (Mch) during de novo heterocyst differentiation, but eventually, the normal pattern partially reappears [23,33]. In contrast, inactivation of *hetN* results in a normal de novo pattern, but Mch formation during successive rounds of differentiation [27]; thus, PatS appears to be responsible for establishing the spatial heterocyst pattern, while HetN is responsible for its maintenance. Addition to the medium of synthetic penta- or hexapeptides (denoted PatS-5 for RGSGR and PatS-6 for ERGSGR) corresponding to the C-terminal (in PatS) or internal (in HetN) motifs inhibits differentiation, but cannot restore a wild-type heterocyst pattern in a *patS* mutant [23,34]. Analysis of *hetN* deletion variants suggested that the RGSGR motif is required for both inhibitory and patterning activity, and thus PatS and HetN regulate different stages of heterocyst patterning utilizing the same amino acid motif for intercellular signaling [26,35]. The actions of PatS and HetN are affected by a third protein, PatA [36], evidently a response regulator, without which *Anabaena* PCC 7120 produces only terminal heterocysts [17,37].

Besides negative regulators, a positively acting factor, HetL, may be involved in HetR regulation. It is a member of a large family of poorly characterized pentapeptide repeat proteins abundant in cyanobacteria [38] and is composed of 40 pentapeptides (A(D/N)LXX). On a multicopy plasmid *hetL* restored the ability of the PatS-overexpressing strain to differentiate heterocysts, while ectopic overexpression in wild type induced Mch in a nitrate-containing medium [39]. However, inactivation of *hetL* did not impair heterocyst development and diazotrophic growth [33]. A recent publication [40] sheds light on the interplay between HetL, HetR, PatS, and PatX, and provides evidence that HetR interacts with HetL at the same interface as PatS and PatX, but without inhibiting its DNA binding activity, suppressing inhibition of heterocyst differentiation. HetL competes with PatS and PatX for HetR binding, and thus it acts as a competitive activator of HetR, complicating its regulation.

We showed previously that the *hetR* gene arose in filamentous cyanobacteria, likely for the regulation of patterned differentiation of specialized cells, long before they learned how to secure nitrogenase in microoxic heterocysts, and it was invariably accompanied by *patX* and no other RG(S/T)GR-containing regulator protein [16]. It is not alone, however, in *Anabaena* PCC 7120, one of a small a group with three RG(S/T)GR-containing negative regulators of HetR [16]. It is therefore of interest to determine the role PatX plays along with PatS and HetN in regulating heterocyst differentiation. Here, we present the results of mutational analyses of the three RG(S/T)GR-containing proteins in *Anabaena* PCC 7120, alone and in combinations, under conditions that promote heterocyst differentiation, and show that unrestrained HetR activity was lethal under different growth conditions. In a nitrogen-depleted medium, PatX had partially overlapping functions with PatS, but all three negative regulators showed different lesions in intercellular signaling. We attribute these apparently contradictory results to interference of RG[S/T]GR-containing negative regulators with a competitive activator HetL due to a rivalry for HetR binding at the same interface. We also show that in a nitrogen-replete medium, PatX was required in the absence of PatS and PatS was required in the absence of PatX for pattern formation, irrespective of the presence or absence of HetN.

## 2. Materials and Methods

### 2.1. Strains, Growth Conditions and Microscopy

*Anabaena* PCC 7120 was grown in nitrate-replete BG-11 (N+) medium or in BG-11_0_ medium free of combined nitrogen (N-) [41] and, when appropriate, free of copper (Cu-; for regulation of the *petE* promoter), at 30 °C illuminated by cool white fluorescent light at 2000 l× (ca. 28 µmol photons/m^2^/s). For ammonium-containing medium, NH_4_Cl (2.5 mM) and MOPS buffer (5 mM, pH 8.0) were added to BG-11_0_. Single and double recombinants and strains carrying replicative plasmids were grown in the presence of appropriate antibiotics at the following final concentrations: neomycin, 25 µg/mL for solid medium and 15 µg/mL for liquid medium; erythromycin, 5 µg/mL; spectinomycin, 5 µg/mL plus streptomycin, 2.5 µg/mL.

To induce heterocyst formation in liquid medium, the filaments from fresh streaks on BG-11 plates were transferred with sterile toothpicks into a 96-well microtiter plate containing liquid BG-11_0_ medium. Cells were routinely examined by bright-field microscopy with an Axiostar plus microscope (Zeiss International, Jena, Germany) equipped with an EOS 1300D(W) digital camera (Canon Inc., Tokyo, Japan), using 40× objective magnification. To visualize the heterocyst-specific envelope polysaccharide layer, a 0.5% Alcian blue solution in 50% ethanol was used for staining cyanobacterial filaments before microscopic examination [42].

### 2.2. Plasmid and Strain Construction

The strains and plasmids used in this study are listed in Table 1. *patX* mutant strains RIAM1238 (DR929; *patX*::Ω; Sm^r^/Sp^r^) and RIAM1239 (DR931; Δ*patX*::Ω; Sm^r^/Sp^r^) were constructed as follows. A 2.9 kb SalI-XmnI fragment containing the 3′-end of *alr2333*, *asl2332* (*patX*), *alr2331, asl2329,* and the 3′-end of *alr2328* from anp03869 was ligated between the SalI and SmaI sites of pK18, producing pRIAM780, and an Ω cassette (a Sm^r^/Sp^r^ determinant flanked by transcriptional terminators) was excised with SmaI from pAM684 and inserted into the internal ScaI site of *patX* in pRIAM780. A construct was selected with the orientation of *aadA* (Sm^r^/Sp^r^) parallel to *patX*, producing pRIAM796.

Plasmid pRIAM780 containing the 3′-end of *alr2333*, *asl2332* (*patX*), *alr2331*, *asl2329* and the 3′-end of *alr2328* derived from anp03869 was digested with ScaI and DraI, deleting most of *patX* and its 3′ UTR, replacing the lost sequence with an Ω cassette excised with SmaI from pAM684. A construct was selected with the orientation of *aadA* (Sm^r^/Sp^r^) parallel to *patX*, producing pRIAM917. Plasmid pRIAM860 is a 4.65-kb derivative of anp03226 with the sequence between AfeI and SmaI sites deleted, leaving all of *all2333*, *asl2332* (*patX*), *alr2331*, *asl2329*, and the 3′-end of *alr2328.* The plasmid was cut with KpnI and partially with BspEI and ligated with pRIAM917 and pRIAM796 digested with KpnI + BspEI, producing pRIAM923 and pRIAM925, respectively. Finally, inserts from pRIAM925 and pRIAM923 were excised with SacI+PstI and placed between the same sites of suicide vector pRL271, producing pRIAM929 and pRIAM931, respectively. All steps of the constructions of pRIAM929 and pRIAM931 are depicted in Supplemental Figure S1.

To delete *patS* from the *Anabaena* PCC 7120 chromosome, an insert from pAM1035 was excised with BamH + SalI and moved to pK18, producing pRIAM1159. A C.CE3 Cm^r^ Em^r^ cassette was excised with EcoICRI from pRL1567 and inserted between the EcoRV and ScaI sites of pRIAM1159, replacing a *patS*-bearing 0.38 kb chromosomal fragment with Cm^r^ Em^r^ genes parallel to excised *patS*, producing pRIAM1175. The insert from this plasmid was moved as a SacI-PstI fragment between the same sites of suicide vector pRL278, producing pRIAM1177. All steps of the constructions of pRIAM1177 are depicted in Supplemental Figure S2.

The plasmids pRIAM929, pRIAM931, and pRIAM1177 were transformed into *E. coli* strain AM1359 [23] containing a broad host range plasmid pRL443 with conjugal functions and a pRL623 plasmid to provide both methylation and mobilization functions and then conjugated into *Anabaena* PCC 7120, its derivative strain PN, and other successive mutant derivatives as indicated in Table 1, using standard protocols [51]. The next day, conjugation plates were underlaid with appropriate antibiotics to select for single recombinants. Subsequent selection for double recombinants using the *sacB* gene present on the vectors was performed as described by [52]. After multiple rounds of successive cloning, the genotypes of all constructed mutant strains were confirmed by PCR analysis (Supplemental Figure S3).

## 3. Results

### 3.1. Mutational Analysis Suggests That in Nitrogen-Depleted Medium, patX is Impaired in Cell–Cell Signaling and Acts Cell-Autonomously

**Figure 1:**
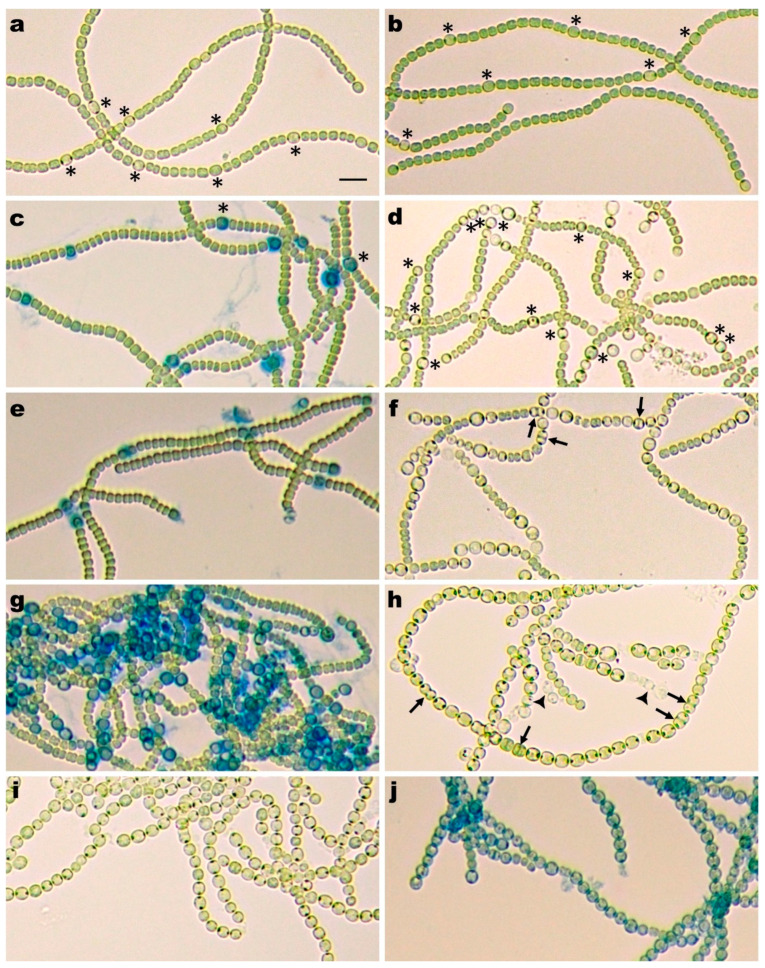
Phenotypes of the wild-type and mutant strains after transfer from a BG-11 plate to liquid BG-11_0_ copper-free (Cu-) medium. Strains were either unstained (**a**,**b**,**d**,**f**,**h**,**i**) or stained with Alcian Blue (**c**,**e**,**g**,**j**) to identify differentiating cells or mature heterocysts. (**a**) Wild-type *Anabaena* PCC 7120 and (**b**) ∆*patX*::Ω single mutant RIAM1239, 1 day after transfer; (**c**) mutant strain PN P*_petE_*-*hetN*, 1 day after transfer; (**d**,**e**) P*_petE_*-*hetN* ∆*patX* conditional double mutant RIAM1242, (**d**) 4 days or (**e**) 1 day after transfer; (**f**,**g**) P*_petE_*-*hetN* ∆*patS* conditional double mutant RIAM1250, (**f**) 2 days or (**g**) 1 day after transfer; (**h**) Δ*patS* Δ*patX*::Ω*(*pAM1714) conditional double mutant RIAM1243, 3 days after transfer; (**i**,**j**) *hetN patX patS* conditional triple mutant RIAM1248, (**i**) 2 days or (**j**) 1 day after transfer. Some heterocysts in panels (**a**) through (**d**) are indicated by asterisks. Apparently dividing aberrant heterocysts in panels (**f**) and (**h**) are indicated by arrows, necridia in (**h**) by arrowheads. The bar in panel (**a**) represents 10 μm.

PatX shares the ability of PatS and HetN to suppress heterocyst differentiation when overexpressed [16]. The phenotypes of *patS* and *hetN* single and double mutants were previously described in detail. The *patS* deletion single mutant strain UHM114 [43] and *patS* replacement mutant [23,33] produce both single heterocysts and Mch in nitrogen-depleted (N-) medium. A conditional P*_petE_*-*hetN* mutant transferred into copper-free (Cu-) N- medium initially displays the wild-type pattern, but later, Mch starts to appear [27], while a ∆*hetN* strain CSL7 forms Mch without lag [35]. Unlike the *patS* and *hetN* mutants, inactivation of *patX* did not cause any visibly aberrant phenotype in nitrogen-depleted (N-) medium. The mutant strains RIAM1238 (*patX*::Ω) and RIAM1239 (∆*patX*::Ω) mutant strains behaved similarly to each other. Qualitatively, both the time course of differentiation and heterocyst pattern were essentially the same as in the wild-type strain (Figure 1a,b). Since the seemingly normal phenotype of *patX* mutants growing in N- medium could be caused by a functional redundancy or impaired function of PatX, we attempted to construct and examine the phenotypes of strains with different combinations of *patX*, *patS*, and *hetN* mutations.

It was previously reported that inactivation of *hetN* results in an unstable Mch phenotype tending to change to a Het^−^ phenotype upon extended subculturing [24]. To avoid the instability, we exploited an approach used previously by [27], who constructed strain 7120PN in which the coding region of *hetN* was fused to the *petE* promoter controlled by the level of copper. At the concentration of copper in BG-11 medium (0.3 µM), the ectopic expression of *hetN* from this promoter is sufficient to completely suppress heterocyst differentiation in *Anabaena* PCC 7120, while in Cu- medium residual *hetN* expression is low enough to virtually eliminate the HetN-mediated suppression of HetR activity [27,43]. The phenotype of a ∆*patS* derivative of 7120PN was described earlier [43].

In order to assess whether the functional role of PatX is masked by PatS and HetN, we made use of the original conditional mutant strain 7120PN (P*_petE_*-*hetN*) [27] with *patX* knocked out: RIAM1241 (P*_petE_*-*hetN patX*::Ω) and RIAM1242 (P*_petE_*-*hetN* Δ*patX*::Ω). In addition, we looked at the phenotypes of two strains similar to the previously reported 7120PN **∆***patS* mutant UHM100 [43]: RIAM1249 and RIAM1250 (independent isolates of P*_petE_*-*hetN* Δ*patS*::C.CE3). Both Δ*patS* mutants showed identical phenotypes, similar to that of UHM100, but different from 7120PN ∆*patX*.

In N^−^ Cu^−^ medium, RIAM1241 and RIAM1242 behaved exactly like the parental strain, the *patX^+^ hetN* conditional mutant 7120PN: one day after induction, a single semiregular heterocyst formed (Supplemental Figure 1c,e), but during consecutive rounds of differentiation, Mch started to appear due to the formation of new heterocysts adjacent to existing single or multiple heterocysts (Figure 1d). 

Transfer of RIAM1250 (P*petE*-*hetN* Δ*patS*::C.CE3) filaments in liquid N^−^ Cu^−^ medium resulted in a deteriorated heterocyst pattern with production of a Mch pattern at 24 h (Figure 1g) and progressive ectopic differentiation upon further incubation (Figure 1f), so that nearly complete differentiation occurred after 4–5 days. There was no growth and cell sediment bleached. This behavior was essentially the same as described earlier for a strain UHM100, also lacking *patS* and conditional in *hetN* expression [43]. In the case of our double and triple mutants, the relative abundance of aberrant heterocysts varied in particular mutants and was most prominent in RIAM1243 (Δ*patS* Δ*patX*::Ω*(*pAM1714)) (ca. 20%, by visual inspection), perhaps due to the presence of a functional copy of *hetN,* which could influence the activity of HetR at late stages and somehow compromise the block of division.

Our initial attempts to construct a Δ*patS* Δ*patX*::Ω double mutant failed: while single recombinants were easily obtained, positive selection for double recombinants [52] on sucrose plates produced only very rare colonies. All retained the Em^r^ marker of the pRL271 suicide vector, indicating the absence of gene replacement by double recombination. However, after conjugation of a P*_petE_*-*patS*-containing plasmid pAM1714 marked by Nm resistance [23] into the Δ*patS* SR931/pRL271 meridiploid single recombinants (containing both the WT *patX* and Δ*patX*::Ω genes), which is viable and has a Δ*patS* phenotype, we obtained many Em^s^ Nm^r^ colonies on sucrose plates, indicating double recombination. A completely segregated conditional double mutant, RIAM1243 (Δ*patS* Δ*patX*::Ω*(*pAM1714)), bleached upon transfer to Cu- medium and, apparently, stopped growing, even in nitrate-containing medium. Massive synchronous heterocyst differentiation was visible after 1–2 days in N- medium (Figure 1h), single necridia were rare in some filaments, but formed multiple contiguous necridia in others, and filaments of differentiated cells contained abundant aberrant (pro)heterocysts with mid-cell envelope constrictions and/or division planes.

A triple mutant RIAM1248, defective in *patS*, in *patX*, and conditionally in *hetN*, was constructed, with viability maintained by *hetN* expression from a copper-regulated promoter. As with the *patS patX* double mutant (see above), transfer of the triple mutant into N- Cu- medium resulted in synchronous ectopic differentiation at 24 h (Figure 1j). After 48 h, filaments showed little fragmentation and consisted almost exclusively of proheterocysts and heterocysts (Figure 1i). Aberrant (apparently dividing) heterocysts were comparatively rare in the triple mutant.

### 3.2. In Nitrogen-Replete Medium, Either patS or patX Alone Can Promote Semiregular Pattern Formation

**Figure 2:**
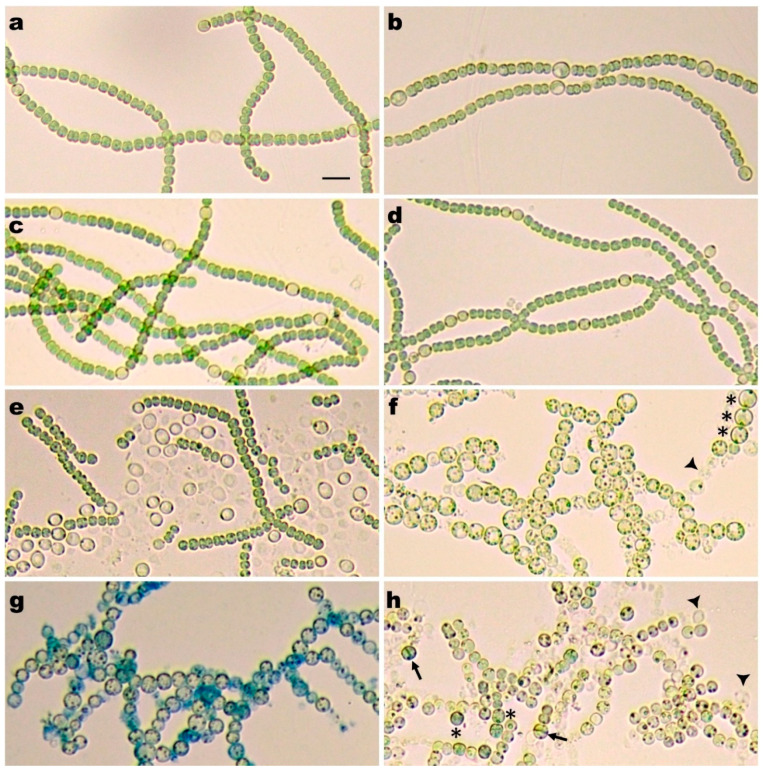
Phenotypes of mutants after transfer from a BG-11 plate into liquid copper-free (**a**,**c**–**h**) nitrate-containing or (**b**) ammonium-containing BG-11 medium. Strains were either unstained (**a**-**f**,**h**) or stained with Alcian Blue (**g**). (**a**,**b**) ∆*patX*::Ω single mutant RIAM1239, 1 day after transfer; (**c**) P*_petE_*-*hetN* **∆***patX* conditional double mutant RIAM1242 and (**d**) P*_petE_*-*hetN* **∆***patS* conditional double mutant RIAM1250, (**d**) 2 days or (**e**) 6 days after transfer; (f,**g**) Δ*patS* Δ*patX*::Ω*(*pAM1714) conditional double mutant RIAM1243, 3 days after transfer; (**h**) *hetN patX patS* conditional triple mutant RIAM1248, 4 days after transfer. Some cells resembling heterocysts in panels (**f**,**h**) are indicated by asterisks and aberrant round cells with division planes in panel (**h**) by arrows. Some necridia (cell ghosts) are marked in panels (**f**,**h**) with arrowheads. The bar in panel (**a**) represents 10 μm.

On BG-11 plates, RIAM1239 (defective in *patX*) did not form heterocysts; however, unlike the wild type, the mutant strain upon transfer from solid to liquid nitrate- or ammonium-containing BG-11 medium produced semiregular heterocysts in many filaments, although at a lower frequency than when transferred from solid BG-11 to liquid N^−^ medium (Figure 2a,b). Differentiation of heterocysts in nitrogen-replete (N+) medium resembles formation of constitutive heterocysts by *patS* and *hetN patS* mutants [23,33,36,43].

Upon transfer to N^+^ Cu^−^ medium, the *hetN* **∆***patX* conditional mutant RIAM1242 formed semiregular predominantly single heterocysts (Figure 2c), and the *hetN* ∆*patS* conditional mutant RIAM1250 in N^+^ Cu^−^ medium behaved similarly to RIAM1242—it also formed semiregular single and some double heterocysts (Figure 2d). However, 5–6 days after the transfer, RIAM1250 started to fragment, first at vegetative cell-heterocyst junctions. Further incubation resulted in progressive fragmentation to very short filaments, single vegetative cells, and detached heterocysts (Figure 2e). Evidently, both PatS and PatX are necessary to restrain HetR sufficiently to prevent differentiation in N+ liquid medium, while only one of them is necessary to produce a semiregular pattern of heterocysts.

A double mutant defective in both *patS* and *patX* exhibited a more extreme phenotype under N+ conditions. The Δ*patS* Δ*patX*::Ω*(*pAM1714) conditional double mutant RIAM1243 grew as filaments of green vegetative cells on BG-11, but upon transfer into N^+^ Cu^−^ liquid medium, fragmented into chains of bleached enlarged round cells with small cyanophycin-like granules in the cytoplasm as well as single and multiple contiguous necridia (Figure 2f). Most of the enlarged round cells morphologically did not resemble heterocysts, but a few of them had a thicker envelope and polar nodules similar to those located in the neck regions of heterocysts (Figure 2f, indicated by asterisks). Apparently, in N+ medium, the differentiation process is induced by transfer into liquid BG-11, but is disorganized and in most cases ceases soon after the initial and early stages. These stages include disintegration of thylakoids and loss of pigmentation, and deposition of the additional outer polysaccharide layer as indicated by Alcian blue staining (Figure 2g).

The RIAM1248 triple mutant behaved differently from the RIAM1243 double mutant. After transfer to N+ Cu- medium, the induced differentiation process was also disorganized but proceeded more slowly, and during the first two days, many cells retained their shape and size but gradually lost pigmentation, and necridia-like cells appeared. After 4 days, the filaments bleached and fragmented due to massive necridia formation, and some enlarged pale-green round cells with thickened envelopes and division planes were visible but without any sign of envelope constriction. Cells with colorless transparent cytoplasm with large granules were also seen (Figure 2h). Both RIAM1243 and RIAM1248 produced multiple necridia looking like cell ghosts, which increased in number during prolonged incubation (Figure 2f,h).

### 3.3. Instability of Conditional Mutants Overexpressing patS or hetN

In the course of our experiments with conditional mutants, we observed very high mutation rates of strains overexpressing either *patS* or *hetN*. These strains were constructed to conditionally block differentiation and thus avoid lethality or eliminate a detrimental burden of extra heterocyst production during cultivation. Although this approach permitted us to obtain and document the phenotypes of strains with multiple mutations, working with such mutants was tricky and required constant microscopic control and frequent cloning to prevent otherwise rapid changes in mutant phenotypes.

**Figure 3:**
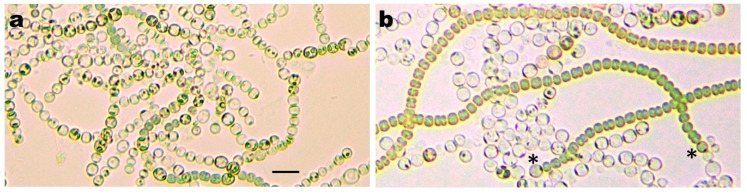
Phenotypes of Δ*patS* Δ*patX*::Ω(pAM1714) conditional double mutant RIAM1243 after transfer from a BG-11 plate into liquid copper-free nitrate-free (Cu- N-) BG-11_0_ medium and incubation for 14 days. (**a**) A freshly isolated RIAM1243 clone and (**b**) the same mutant after several subcultures on BG-11+Sp, Sm, Nm plate. Asterisks in panel (**b**) indicate terminal heterocysts. The bar in panel (**a**) represents 10 μm.

Several passages on BG-11 plates with or without appropriate antibiotics of fully segregated conditional mutants resulted in a rapid accumulation of secondary mutations. For example, Figure 3 shows a double mutant strain RIAM1243 before and after several subcultures on selective BG-11 + Sp, Sm, Nm plates and subsequent transfer and incubation in N^−^ Cu^−^ medium for 2 weeks. While a freshly isolated RIAM1243 clone after prolonged incubation exhibited only rare short stretches of vegetative cells originating from rare single cells that remained undifferentiated (Figure 3a), long filaments of vegetative cells (Het^−^ phenotype), sometimes with terminal single or multiple heterocysts (PatA-like or PatA Mch phenotypes), accumulated in suspensions of fragmented chains of heterocysts (Figure 3b). Similar instability was exhibited by the conditional P*_petE_*-*hetN* Δ*patS* mutant RIAM1250 (Figure 2e) and the triple mutant RIAM1248 (not shown).

There was no growth in both cases, and both cultures contained a light yellowish-grey sediment. We used in experiments only freshly isolated clones with all filaments showing similar phenotypic characteristics.

## 4. Discussion

PatX is the only RG(S/T)GR protein whose association with HetR is more ancient than the heterocyst-forming cyanobacteria [16]; however, its function remains obscure. Prior to this work, all that was known was that ectopic overexpression of PatX blocked heterocyst differentiation in *Anabaena* PCC 7120 [16], and so did PatX orthologs from *Mastigocladus laminosis* [53] (called “alternative PatS”) and from *Arthrospira platensis* NIES 39 [54] (called “PatS”), a filamentous strain that does not form heterocysts. To clarify the role of PatX in heterocyst regulation, we disrupted the *patX* gene, but the resulting mutant strain grown in the absence of a nitrogen source showed no heterocyst-related phenotype. This could be a consequence of functional redundancy or a functionality unrelated to heterocyst formation. Loss of PatX also produced no obvious phenotypic change in combination with a conditional *hetN* mutation. Under conditions in which *hetN* was not expressed, two mutant strains, one carrying conditional *hetN* and the other identical but with *patX* knocked out, displayed a similar delayed Mch phenotype (Figure 1d). Both strains were phenotypically different from the conditional *hetN patS* double mutant UHM100 described previously [36,43] and from an analogous double mutant RIAM1250 constructed in this work, which showed gradual asynchronous differentiation so that in a few days, almost all cells became heterocysts (Figure 1f). These results demonstrate that the functionality of *patX* differs from that of *patS*. PatS is required for de novo pattern formation, but is not sufficient to prevent later formation of contiguous heterocysts (Mch). PatX cannot replace PatS in directing de novo pattern under nitrogen-depleted (N-) conditions, nor can it replace HetN in maintaining a pattern.

A residual functionality of *patX* becomes obvious on comparison of a ∆*patS* mutant with a ∆*patS* ∆*patX* double mutant RIAM1243, both retaining a wild-type *hetN* (Figure 1, [23]). The double mutant is inviable unless *patS* is conditionally suppressed by placing the gene under the control of the copper-regulated *petE* promoter. Upon removal of copper from the medium, this double mutant behaved essentially as the conditional *hetN patX patS* triple mutant RIAM1248—both started nearly synchronous differentiation of all vegetative cells into heterocysts when incubated in N^−^ Cu^−^ medium (Figure 1h–j). The intact chromosomal copy of *hetN* in RIAM1243 could not prevent or slow down the differentiation process. Thus, PatX (along with PatS) is required to prevent rapid synchronous differentiation of all vegetative cells.

These results indicate that upon combined nitrogen deprivation, all three negative regulators are instrumental in promoting regular pattern formation and/or maintenance, but in different ways. The roles of PatS and HetN in pattern initiation and maintenance, respectively, are readily discernible, but the role of PatX is masked by the presence of PatS. Our results are in line with those of Risser and Callahan [20], who observed the formation of concentration gradients of HetR in proximity to heterocysts, dependent on either *patS* or *hetN*. When both genes were deleted, no HetR gradients formed, indicating that either *patS* or *hetN* is required for establishing HetR concentration gradients after combined nitrogen removal. Their results demonstrate that *patX* (unknown at the time) is unable to induce HetR gradient formation in the absence of *patS* and *hetN* and thus does not participate in cell–cell signaling in the same way as *patS* and *hetN*.

A role for PatX in regulating heterocyst differentiation is more easily seen when *Anabaena* PCC 7120 is grown in nitrogen-replete (N+) medium. Unlike the wild-type strain, the *patX* mutant RIAM1239 produced morphologically distinct semiregular heterocysts when transferred to liquid nitrate or ammonia-containing media (Figure 2a,b), indicating that its product was needed to prevent unnecessary differentiation. Conditional double mutants RIAM1242 (*hetN patX*) and RIAM1250 (*hetN patS*) also formed semiregular heterocysts in copper-free (Cu-) medium (Figure 2c,d), indicating that in N+, either PatS or PatX is sufficient for rapid pattern formation. At the same time, both conditional double mutant RIAM1243 (*ΔpatS ΔpatX*::Ω(pAM1714)) and conditional triple mutant RIAM1248 (*hetN patX patS*), after removal of copper, started ectopic aberrant differentiation. With both mutants, all cells differentiated into morphologically distinct heterocysts in N- Cu- medium, but in N+ Cu- medium, differentiation into heterocyst-like cells was rare, and aberrant differentiation led to the appearance of enlarged spherical cells that partially retained pigmentation and of necridia, resulting from cell lysis (Figure 2f,h). Apparently, unrestrained HetR in N+ medium promotes differentiation that is misdirected. The low level in N+ medium of NtcA and/or its activator 2-oxoglutarate [55] could exacerbate the situation.

**Figure 4:**
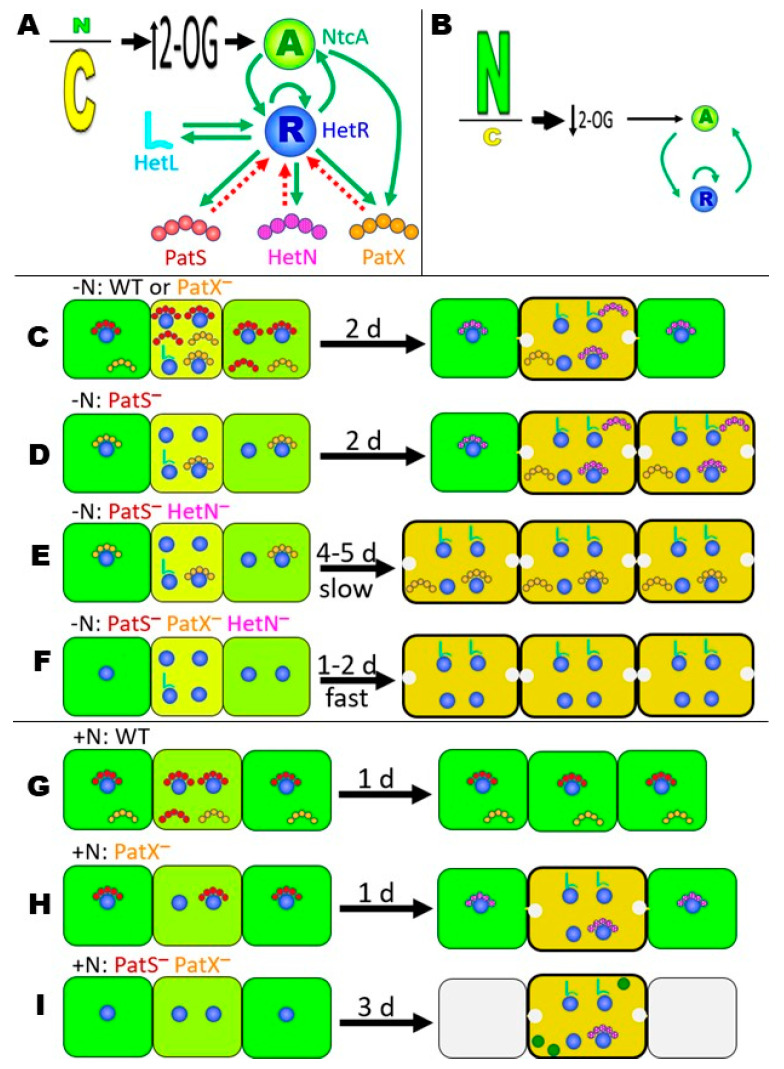
Model integrating roles of different signals in differentiation that are considered in this work. (**A**,**B**) Major actors under conditions of low nitrogen (N) and high carbon (**C**) or the reverse. Green solid arrows indicate an increase in transcription and/or activity, while red dotted arrows indicate repression of either transcription and/or activity. 2-OG represents 2-oxoglutarate. (**C**–**F**) The state of three adjacent cells after nitrogen stepdown (-N). Cells that are internally nitrogen-replete are represented as dark green, those that are mildly starved as light green, those that have passed the threshold for differentiation as yellow-green, and differentiated cells as brown with a thick envelope and white polar plugs. The level of HetR is crudely represented by the number of blue spheres, in some cases bound to PatS, HetN, PatX, or HetL (see their icons in panel **A**). (**G**–**I**) The state of three adjacent cells under nitrogen-replete (N+) conditions. The conventions are the same as in panels **C**–**F**. In addition, aberrant heterocysts are shown with the green inclusion bodies seen in Figure 2f,h, and necridia are represented as gray cells.

The interactions between the different signaling molecules discussed here can be visualized through the speculative model shown in Figure 4, one of many possible that fit the data. For some other aspects of signaling, see Flores et al., (2019) [17]. Figure 4A introduces the actors considered in the model and their icons, illustrating that a low N/C ratio increases the level of 2-oxoglutarate, inducing both the activity and transcription of NtcA [55]. NtcA and HetR mutually increase the other’s transcription, forming a positive feedback loop [18], and a high level of HetR (possibly modified, possibly indirectly) increases the transcription of *patS* [56], *hetL* [40], *patX* [29], and eventually *hetN* [28,29]. Some form of PatS, PatX, and presumably HetN negatively affect the activity of HetR [17,40], while HetL protects HetR from its inhibitors [40]. The model represents the three RG(S/T)GR proteins as five circles, but this is not intended to imply that the pentapeptide is the natural signal within *Anabaena* PCC 7120 [26,35,40,57,58]. Here we refer to the active signal loosely as PatS, PatX, and HetN. Under N+ conditions (Figure 4B), the level of 2-oxoglutarate is reduced, leading to lower levels and activity of NtcA and HetR. PatS is expressed at a low level under these conditions [29,33], and PatX is as well ([29]; Khudyakov, unpublished).

After nitrogen stepdown (-N), cells within wild-type filaments are represented as experiencing different levels of nitrogen starvation [59,60] (Figure 4C, left). N-deprivation increases the level of HetR from a basal level, but the levels of PatS and PatX, whose transcription increases as a result of this change [16,33], are postulated to be sufficient to bind to HetR and inhibit at least part of its activity [17,40]. The level of HetN does not increase during this early period [28]. HetL expression also increases after N-stepdown, probably in differentiating cells [40], and HetL competes with PatS and PatX for binding to HetR [40]. It should be noted that HetL protein is not required for normally spaced heterocysts [39] and the degree to which HetL competes with RG(S/T)GR signals in vivo is speculative [40]. In time, cells in which HetR has escaped inhibition proceed on to form mature heterocysts, while the rest revert back to vegetative cell status (Figure 4C, right). The model shows the presence of HetN and PatX but not PatS in mature heterocysts, reflecting their known time course and levels of transcription and/or protein levels [28,31,61]. The level of HetN in heterocysts and its resulting appearance in adjacent cells is required to maintain the pattern of heterocysts [27]. The state of a wild-type strain is depicted, but phenotypically, a strain defective in PatX behaves the same way in N- medium.

In the absence of PatS, cells that would otherwise differentiate still did, but HetR in adjacent cells that are only mildly starved escaped inhibition, perhaps because of an insufficient RG(S/T)GR signal, leading to MCH (Figure 4D). The inability of the mutation of *patX* (in contrast to *patS*) to affect heterocyst spacing may be due to a difference in the binding to modified HetR in heterocysts of the RGTGR-containing PatX signal relative to RGSGR-containing PatS signal [40]. HetN production restrains more extensive differentiation. In the absence of both PatS and HetN, HetR in cells adjacent to heterocysts is not fully inhibited, and over the course of several days, eventually, nearly all cells differentiate (Figure 4E; [43] and our own observations). In the absence of all three RG(S/T)GR signals (or just PatS and PatX), HetR proteins in both starved and mildly starved cells were not inhibited, and all cells rapidly differentiated (Figure 4F).

In wild-type *Anabaena* PCC 7120, cells grown in nitrate or ammonia did not reach the threshold level of starvation for differentiation (Figure 4G). Even in random cells that are mildly starved, the level of PatS and PatX signal was high enough to titrate the low level of HetR. The presence or absence of HetN did not materially affect the phenotype in N+ medium. In strains lacking PatX (Figure 4H) or PatS (not shown; [23]), the level of RG(S/T)GR signal was no longer high enough to titrate the low level of HetR, and so heterocyst differentiation proceeded even in the presence of nitrate or ammonia. When both PatS and PatX were absent, no early-acting RG(S/T)GR signal was present to restrain HetR, and so, over time, all cells were affected, either by differentiating to aberrant heterocysts or by lysing (Figure 4I).

The developmental chaos that results from *patX patS* and *hetN patS* double mutants produces strong selective pressure for the rapid accumulation of suppressor mutations to escape from lethal terminal differentiation. Similar instability has been seen with strains overexpressing HetR [62] or HetZ and HetP [63] and strains with mutated *hetN* [25]. In our experiments, we tried to avoid this problem by using conditional mutants and maintaining them in a medium that blocked heterocyst development completely and thus presumably eliminated negative selection pressure on mutants overproducing heterocysts [43,63]. Nevertheless, a high mutation rate was still observed, resulting in the rapid accumulation of Het^−^ and PatA-like mutants producing only terminal heterocysts (Figure 3). We know of no studies concerning growth defects in N+ medium in strains affected in HetR function or expression, but HetR may well participate in protein interactions and/or influence expression of genes that are unrelated to heterocyst formation but important for vegetative growth [17]. Its overexpression or inhibition of expression and activity could impose selective pressure for the appearance of suppressor mutations.

In the presence of combined nitrogen, either PatX or PatS as the sole HetR-regulator could establish and maintain a wild-type semiregular heterocyst pattern (Figure 2c,d), and in terms of current models of heterocyst pattern formation, they are fully proficient in cell–cell signaling, a prerequisite for pattern formation. This basic mechanism rests on interactions between two players, HetR and PatX/PatS derivatives, perhaps as modeled by a Turing-like reaction-diffusion mechanism [3], it works irrespective of nitrogen status and is hidden by additional layers of regulation, but can be revealed by mutational analyses. These additional layers may respond to the availability of combined nitrogen or other environmental cues and a set of downstream regulators (which can vary in different strains and complicate regulation) transmitting the patterning signals and converting them into morphogenetic and metabolic changes. We conjecture that this basic mechanism was initially designed by filamentous cyanobacteria for the purpose of dissemination by nonrandom fragmentation of trichomes through necridia formation, and later modified and adopted for patterned heterocyst differentiation.

## Figures and Tables

**Table 1:** Strains and plasmids.

Strain or Plasmid Reference	Derivation and/or Relative Characteristics	Source or
*Anabaena* Strains		
PCC 7120	Wild type	S. Callahan
7120PN	P*_petE_*-*hetN*	[27]
RIAM1238	DR929; *patX*::Ω; Sm^r^/Sp^r^	This study
RIAM1239	DR931; Δ*patX*::Ω; Sm^r^/Sp^r^	This study
RIAM1241	PN DR929; P*_petE_*-*hetN patX*::Ω; Sm^r^/Sp^r^	This study
RIAM1242	PN DR931; P*_petE_*-*hetN* Δ*patX*::Ω; Sm^r^/Sp^r^	This study
RIAM1243	UHM114 DR931 (pAM1714); Δ*patS* Δ*patX*::Ω(pAM1714); Sm^r^/Sp^r^ Nm^r^	This study
RIAM1245	PN DR931 DR1177; P*_petE_*-*hetN* Δ*patX*::Ω Δ*patS*::C.CE3; Sm^r^/Sp^r^ Em^r^	This study
RIAM1248	PN DR931 DR1177; P*_petE_*-*hetN* Δ*patX*::Ω Δ*patS*::C.CE3; Sm^r^/Sp^r^ Em^r^	This study
RIAM1249	PN DR1177; P*_petE_*-*hetN* Δ*patS*::C.CE3; Em^r^	This study
RIAM1250	PN DR1177; P*_petE_*-*hetN* Δ*patS*::C.CE3; Em^r^	This study
UHM114	Δ*patS*	[43]
*Plasmids*		
anp03226	A *patX* (*asl2332*)-bearing bp 2805907 to 2813409 fragment of *Anabaena* PCC 7120 chromosome in the BamHI site of pUC18; Ap^r^	[44]
anp03869	A *patX* (*asl2332*)-bearing bp 2803179 to 2811405 fragment of *Anabaena* PCC 7120 chromosome in the BamHI site of pUC18; Ap^r^	[44]
pAM504	Shuttle vector for replication in *E. coli* and *Anabaena*; Km^r^ Nm^r^	[45]
pAM684	A source of the Sp^r^ Sm^r^ Ω cassette; Ap^r^ Sp^r^/Sm^r^	[46]
pAM1035	*patS* on a 3.3 kb chromosomal fragment in pBluescript II KS(-); Ap^r^	[23]
pK18	pBR322-derived cloning vector; Km^r^	[47]
pRIAM780	A 2.9 kb SalI-XmnI fragment containing 3′-end of *alr233*, *asl2332* (*patX*), *alr2331* and 3′-end of *alr2330* from anp03869 ligated in SalI-SmaI sites of pK18; Km^r^	This study
pRIAM796	Ω cassette inserted into internal ScaI site in *patX* ORF in pRIAM780; Km^r^ Sp^r^/Sm^r^	This study
pRIAM860	anp03226 derivative with an AfeI-SmaI fragment deleted; contains *patX* on remaining 4.65 kb insert; Ap^r^	This study
pRIAM917	pRIAM780 with ScaI-DraI fragment containing most of *patX* ORF and 3′ UTR replaced with Ω cassette; Km^r^ Sp^r^/Sm^r^	This study
pRIAM923	A 4.9 kb chromosomal region (bp 2805907 to 2810809) with ScaI-DraI fragment containing most of *patS* ORF and 3′ UTR replaced with Ω cassette; reconstructed from pRIAM860 and pRIAM917; Km^r^ Sp^r^/Sm^r^	This study
pRIAM925	Same as pRIAM917, but the Ω cassette inserted into into internal ScaI site in *patX* ORF; reconstructed from pRIAM860 and pRIAM796; Km^r^ Sp^r^/Sm^r^	This study
pRIAM929	Insert from pRIAM925 moved into suicide vector pRL271; Cm^r^ Em^r^ Sp^r^/Sm^r^	This study
pRIAM931	Insert from pRIAM923 moved into suicide vector pRL271; Cm^r^ Em^r^ Sp^r^/Sm^r^	This study
pRIAM1159	Insert from pAM1035 moved in pK18 (probably as BamH-SalI fragment); Km^r^	This study
pRIAM1175	C.CE3 Cm^r^ Em^r^ cassette excised with EcoICRI from pRL1567 and inserted into EcoRV-ScaI sites of pRIAM1159, replacing *patS*-bearing 0.38 kb chromosomal fragment; Km^r^ Cm^r^ Em^r^	This study
pRIAM1177	Insert from pRIAM1175 moved into SacI-PstI sites of pRL278; Km^r^ Cm^r^ Em^r^	This study
pRL271	*sacB*-containing suicide vector; Cm^r^ Em^r^	[48]
pRL278	*sacB*-containing suicide vector; Nm^r^/Km^r^	[48]
pRL1567	Source of C.CE3 Cm^r^ Em^r^ cassette; Ap^r^ Cm^r^ Em^r^	[49]
pUC19	pBR322-derived cloning vector; Ap^r^	[50]

## Supplementary Materials

The following are available online at https://www.mdpi.com/2075-1729/10/12/326/s1, Figure S1: Construction of pRIAM929 and pRIAM931 (Δ*patX*), Figure S2: Construction of pRIAM1177 (Δ*patS*), Figure S3: Segregation of mutations.
