# Supplementary material for: Inactivation of Three RG(S/T)GR Pentapeptide-Containing Negative Regulators of HetR Results in Lethal Differentiation of Anabaena PCC 7120
Source: Life (Basel). 2020 Dec 4;10(12):326. doi: 10.3390/life10120326 (PMC7761841; doi:10.3390/life10120326)
Supplement: Supplementary file 1 [file life-10-00326-s001.pdf]

Inactivation of three RG(S/T)GR pentapeptide-containing negative regulators of HetR results in lethal differentiation of *Anabaena* PCC 7120

Supplemental Figures

Supplemental Figure 1: Construction of pRIAM929 and pRIAM931 (Δ*patX*)

Supplemental Figure 2: Construction of pRIAM1177 (Δ*patS*)

Supplemental Figure 3: Segregation of mutations

Supplemental Figure S1. Construction of pRIAM929 and pRIAM931 (Δ*patX*)

A. Insertional inactivation of *patX*

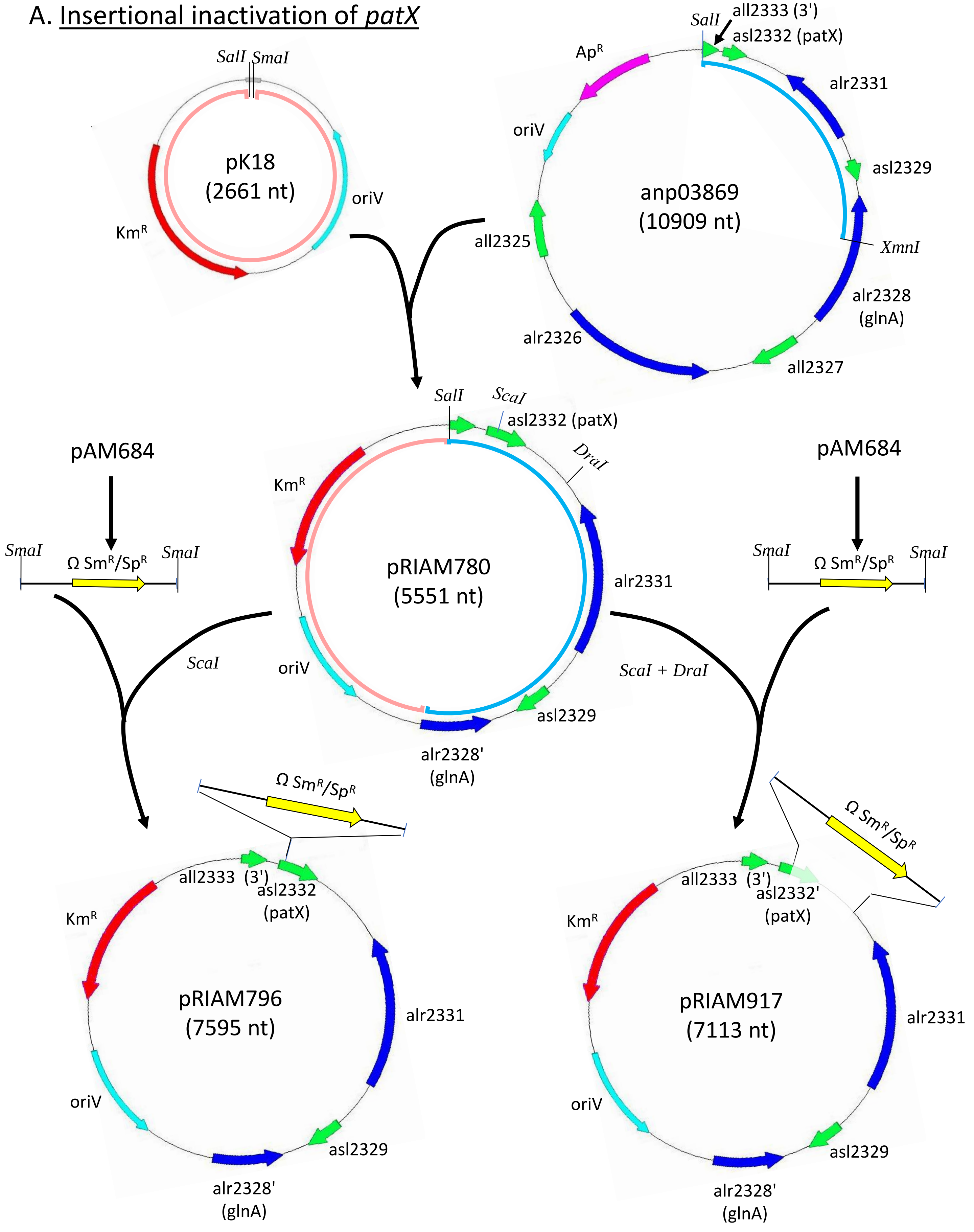

B. Reconstruction of 5' flanking region

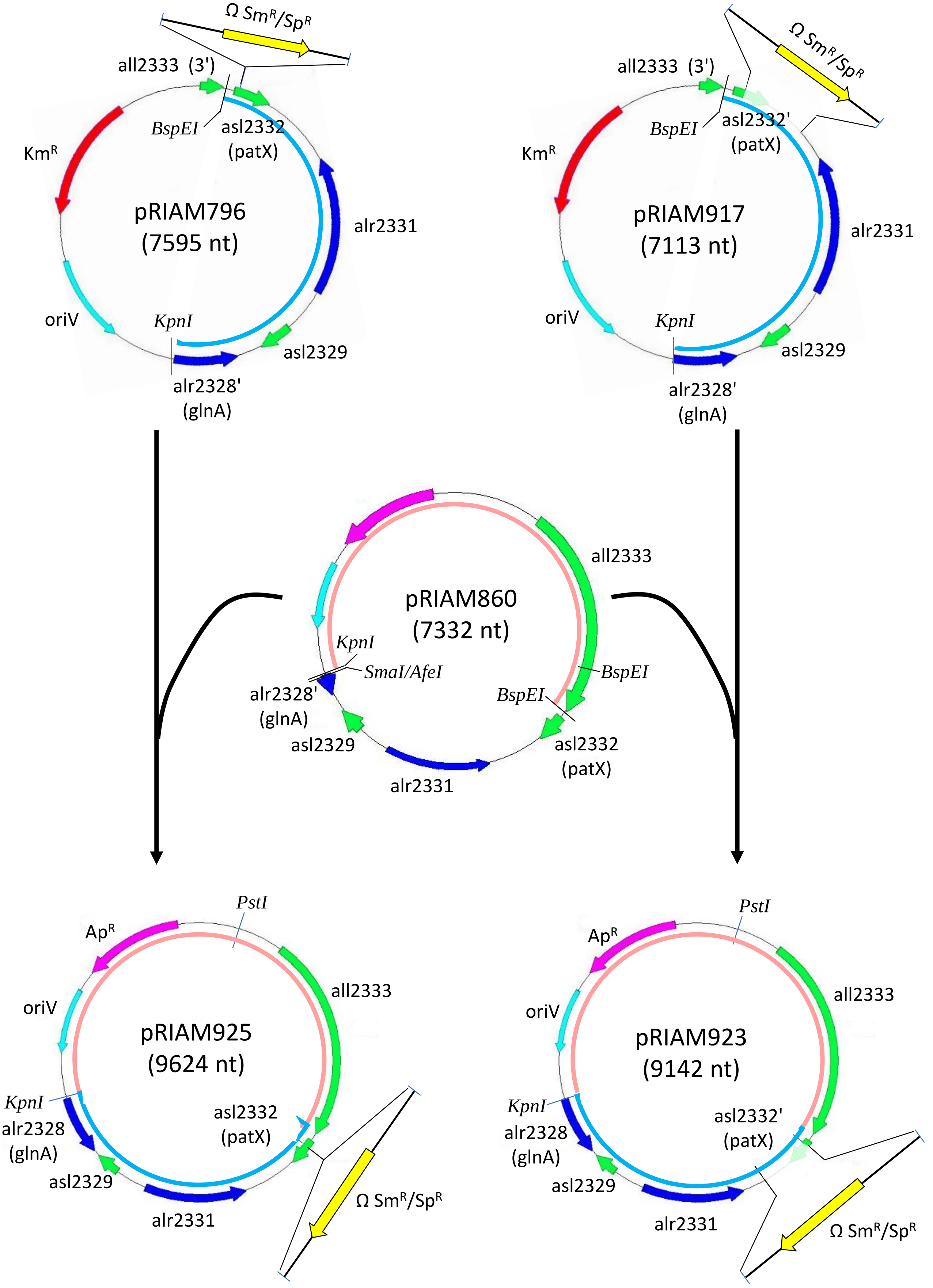

C. Transfer of construct to suicide vector

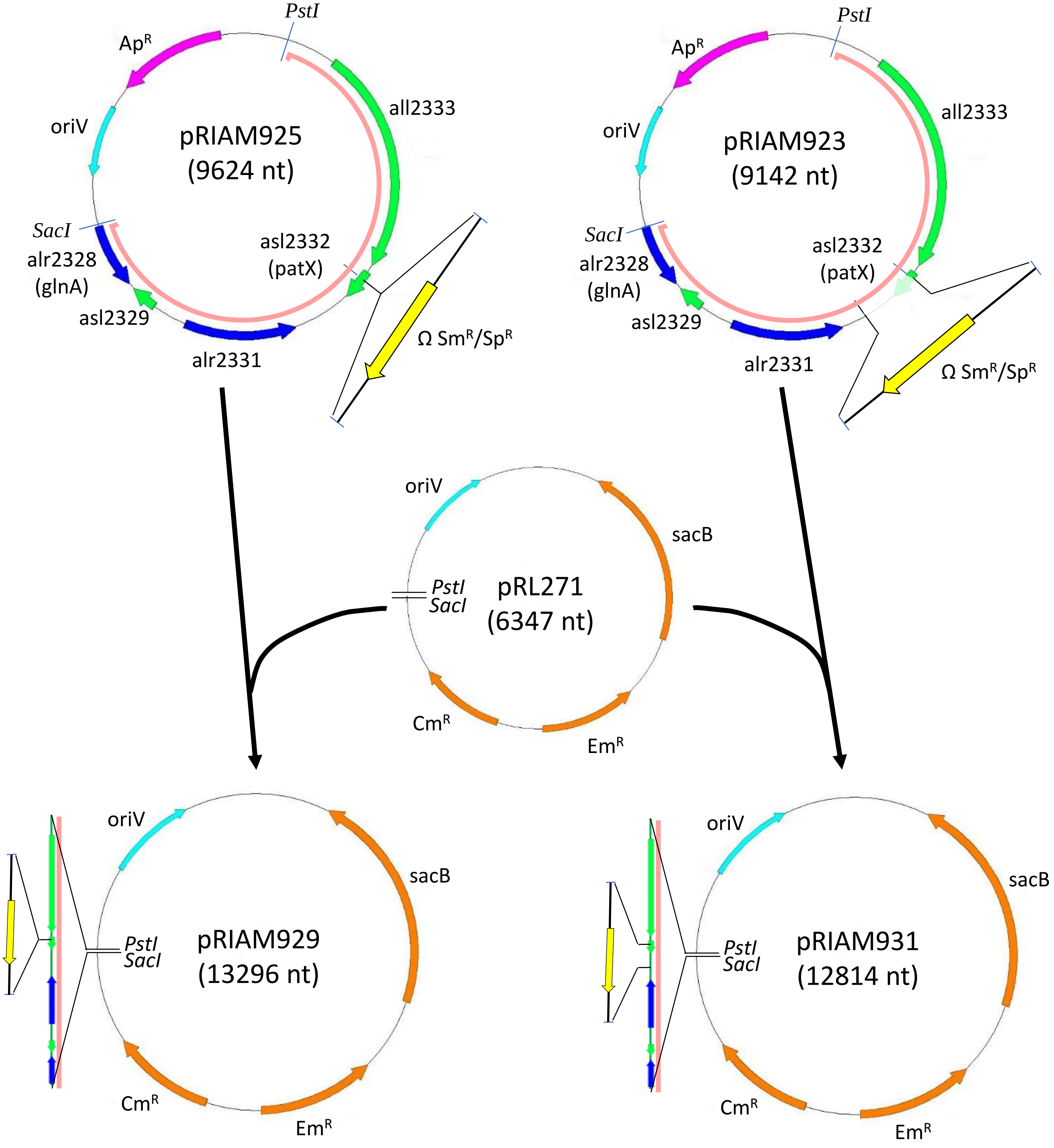

Supplemental Figure S2. Construction of pRIAM1177 (Δ*patS*)

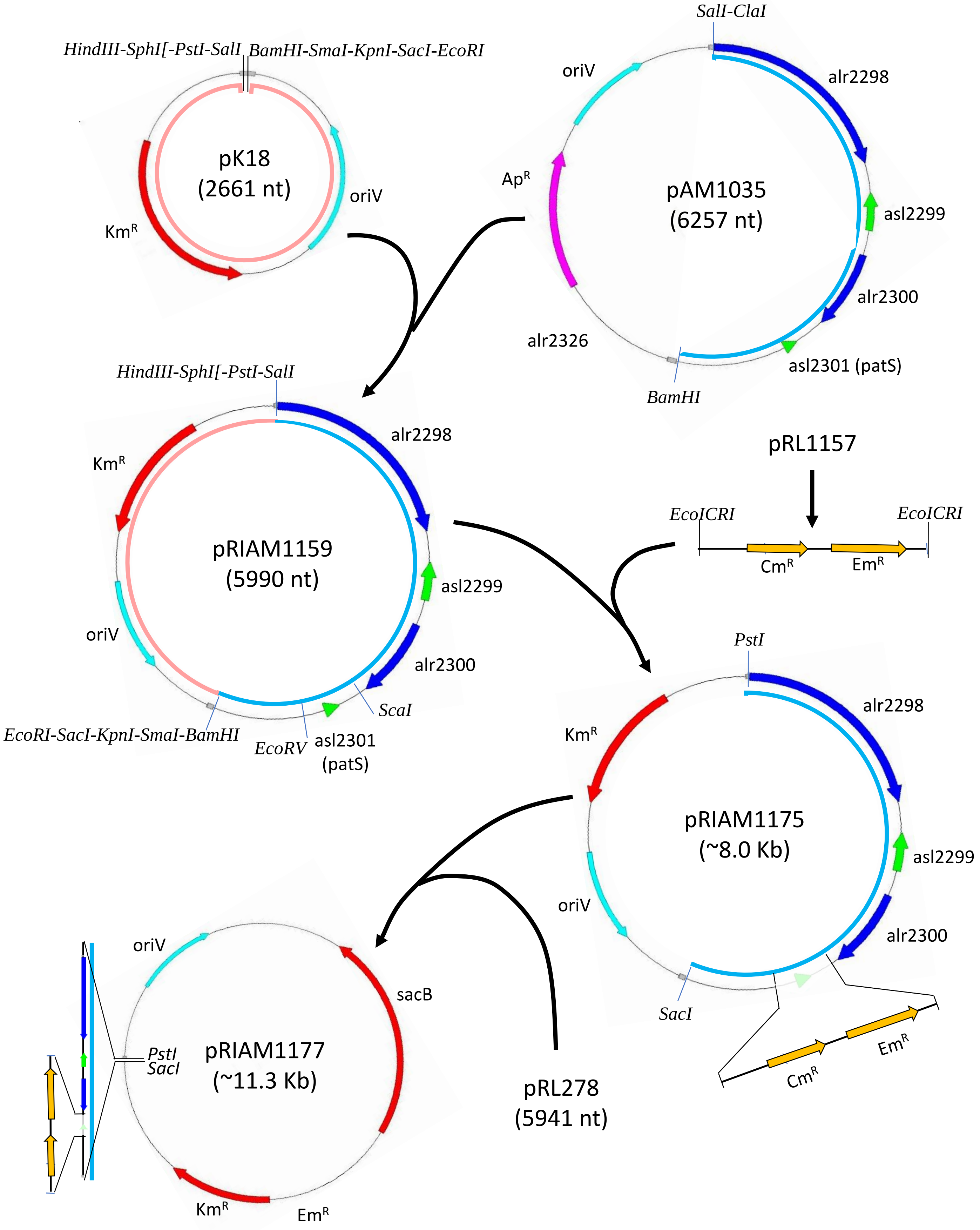

Supplemental Figure S3. Segregation of mutations

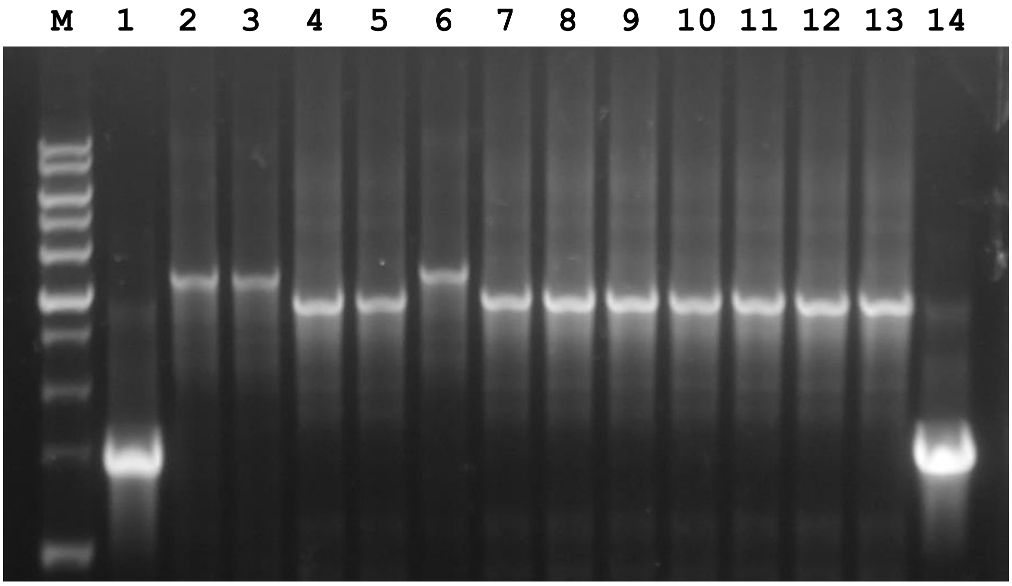

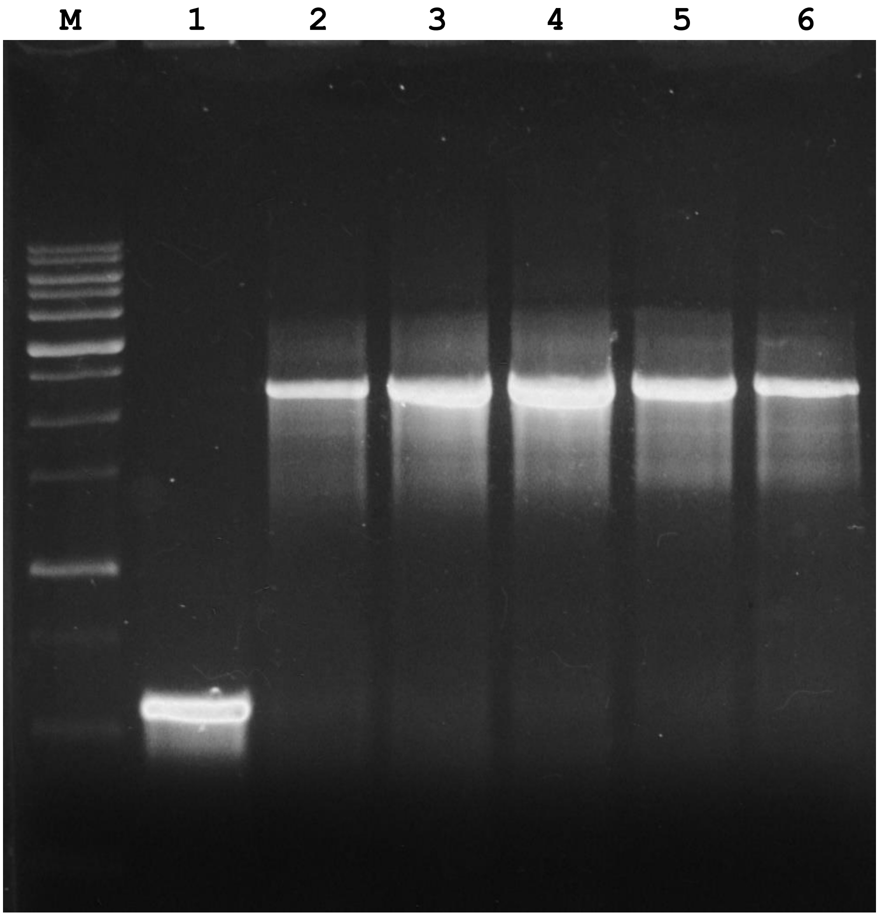

(top) **Segregation of *patX* mutations in constructed *patX* mutant strains.**
Primers 2332-F2 TATTGACACCATGCACACTT and 2331-F1 TCTCAGATGGAGCTCGTCATGCC were used for PCR using chromosomal DNAs from the following strains:

**M**	molecular markers (1kb: 10, 8, 6, 5, 4, **3**, 2.5, 2, 1.5, **1**)
**1**	wild type *Anabaena* PCC 7120
**2,3**	different clones of RIAM1238, patX::Ω
**4,5**	different clones of RIAM1239, Δ*patX*::Ω
**6**	RIAM1241, P_{petE}-*hetN* *patX*::Ω
**7**	RIAM1242, P_{petE}-*hetN* Δ*patX*::Ω
**8,9**	different clones of RIAM1243, Δ*patS* Δ*patX*::Ω(pAM1714)
**10,11**	different clones of RIAM1245, P_{petE}-*hetN* *patX*::Ω Δ*patS*::C.CE3
**13,14**	different clones of RIAM1248, P_{petE}-*hetN* Δ*patX*::Ω Δ*patS*::C.CE3
**15**	wild type *Anabaena* PCC 7120

(bottom) **Segregation of *patS* mutations in constructed *patS* mutant strains.**
Primers 2299-F1 GTCTGCTGTAAGCCTTATCAGC and 2299-R1 CACCATTCAATTGCACCATC were used for PCR using chromosomal DNAs from the following strains:

**M**	molecular markers (1kb: 10, 8, 6, 5, 4, **3**, 2.5, 2, 1.5, **1**, 0.75, 0.5, 0.25)
**1**	wild type *Anabaena* PCC 7120
**3**	RIAM1245, P_{petE}-*hetN* *patX*::Ω Δ*patS*::C.CE3
**3,4**	different clones of RIAM1248, P_{petE}-*hetN* Δ*patX*::Ω Δ*patS*::C.CE3
**5,6**	RIAM1249 and RIAM1250, different clones of P_{petE}-*hetN* Δ*patS*::C.CE3
